# Maximizing CRISPR/Cas9 phenotype penetrance applying predictive modeling of editing outcomes in *Xenopus* and zebrafish embryos

**DOI:** 10.1038/s41598-020-71412-0

**Published:** 2020-09-04

**Authors:** Thomas Naert, Dieter Tulkens, Nicole A. Edwards, Marjolein Carron, Nikko-Ideen Shaidani, Marcin Wlizla, Annekatrien Boel, Suzan Demuynck, Marko E. Horb, Paul Coucke, Andy Willaert, Aaron M. Zorn, Kris Vleminckx

**Affiliations:** 1 Department of Biomedical Molecular Biology, Ghent University, Technologiepark 71, 9052 Ghent (Zwijnaarde), Belgium; 2 Cancer Research Institute Ghent, Ghent, Belgium; 3 Division of Developmental Biology, Perinatal Institute, and Center for Stem Cell and Organoid Medicine (CuSTOM), Cincinnati Children’s Hospital, Cincinnati, USA; 4 Center for Medical Genetics, Department of Biomolecular Medicine, Ghent University, Ghent, Belgium; 5 National Xenopus Resource and Eugene Bell Center for Regenerative Biology and Tissue Engineering, Marine Biological Laboratory, Woods Hole, MA 02543 USA

**Keywords:** Xenopus, Genetic engineering, Genetic techniques, Genetic models

## Abstract

CRISPR/Cas9 genome editing has revolutionized functional genomics in vertebrates. However, CRISPR/Cas9 edited F_0_ animals too often demonstrate variable phenotypic penetrance due to the mosaic nature of editing outcomes after double strand break (DSB) repair. Even with high efficiency levels of genome editing, phenotypes may be obscured by proportional presence of in-frame mutations that still produce functional protein. Recently, studies in cell culture systems have shown that the nature of CRISPR/Cas9-mediated mutations can be dependent on local sequence context and can be predicted by computational methods. Here, we demonstrate that similar approaches can be used to forecast CRISPR/Cas9 gene editing outcomes in *Xenopus tropicalis*, *Xenopus laevis,* and zebrafish. We show that a publicly available neural network previously trained in mouse embryonic stem cell cultures (InDelphi-mESC) is able to accurately predict CRISPR/Cas9 gene editing outcomes in early vertebrate embryos. Our observations can have direct implications for experiment design, allowing the selection of guide RNAs with predicted repair outcome signatures enriched towards frameshift mutations, allowing maximization of CRISPR/Cas9 phenotype penetrance in the F_0_ generation.

## Introduction

Over the last couple of years, CRISPR/Cas9 has revolutionized reverse genetic studies in non-mammalian vertebrate model organisms^1–3^, and has further empowered the use of *Xenopus* and zebrafish as model organisms for studying development and human disease^4–6^. In particular, F_0_ CRISPR/Cas9-mediated gene disruption in non-mammalian vertebrates has emerged as a cost-effective method to rapidly assign causality to genetic variants in candidate disease genes identified from human patient exome sequencing^7–11^. This can assist clinical geneticists in providing timely genetic diagnosis and counseling to patients and affected families, thereby favoring societal and economic impact of findings. CRISPR/Cas9 mediated F_0_ mosaic mutant embryos are also increasingly employed as an alternative to antisense morpholino oligomers (MOs)^12,13^ to investigate gene function and genetic interactions in developing embryos^14^, thus expanding the toolbox for cell and developmental biologists.

An important consideration in CRISPR/Cas9 mutational studies is identifying gRNAs that produce a high frequency of loss-of-function mutations in the appropriate coding exons and hence generate highly penetrant specific F_0_ phenotypes^15^. During gRNA design, considerations include the possibilities of reading frame preservation upon skipping of the targeted exon or translation reinitiation at alternative start codons downstream of the gRNA cut site, culminating in retention of (partially) functional truncated protein variants^16,17^. In coding exons, double strand breaks (DSB) induced by CRISPR/Cas9 reagents are typically repaired by error-prone mechanisms, thereby potentially inducing frameshifting insertions and deletions (INDELs) resulting in premature stop-codons (PTC)^18^. Depending on their position in the primary mRNA, PTCs can subject the resulting transcript to nonsense-mediated mRNA decay (NMD) and effective cellular knockout (KO) of the CRISPR/Cas9-targeted gene can thus be obtained. In contrast, in-frame INDELs in coding exons will lead to the loss or gain of amino acids, which can still result in retention of potentially functional protein variants^19^. We believe that in many cases the inability to retrieve phenotypes in *Xenopus* and zebrafish F_0_ CRISPR/Cas9 edited animals is the consequence of in-frame mutations in a substantial number of cells in the mosaic mutant animal. To circumvent this problem, targeting of gRNA to functional protein domains has been suggested and several tools have been released that allow actively integrating structural information in the gRNA design process^20,21^. However, in-frame mutations in functional domains have the potential to generate dominant (*e.g.* gain-of-function) protein variants. Alternatively, simultaneous targeting of a single gene with multiple gRNAs has been suggested to produce high efficiency loss-of-function^8^, but this method is associated with increased toxicity and magnifies off-target concerns inherent to the CRISPR/Cas9 system.

We, and others, hypothesized that improved gRNAs selection, with editing outcomes enriched for frameshift mutations and NMD, could maximize the penetrance of F_0_ phenotypes^22,23^. It is now accepted that local sequence context surrounding the CRISPR/Cas9-induced DSB dictates editing outcomes and renders them nonrandom^24,25^. DSB repair can be dependent on microhomology mediated end joining (MMEJ), and studies in zebrafish have shown that computational tools can be used to select guide RNAs likely to induce MMEJ^22,26^. In parallel, several research groups have established computational prediction modules based on logistic regression or deep learning to anticipate, in silico, the outcomes of template-free CRISPR/Cas9 editing. Among these are InDelphi, Lindel and FORECasT^27–29^, trained using large datasets of CRISPR/Cas9 editing outcomes in different cellular contexts^30^. Here, we compared experimental outcomes of CRISPR/Cas9 genome editing in *Xenopus* and zebrafish embryos to these publicly available in silico editing outcome prediction models. We find that the InDelphi network that was trained on mouse embryonic stem cells (mESC) is highly predictive for the CRISPR/Cas9 editing outcomes in developing vertebrate embryos. We rationalize this will allow to select gRNAs favoring frameshift gene editing outcomes prone to nonsense-mediated decay (NMD), thereby maximizing subsequent protein knock-out in CRISPR/Cas9 animal models.

## Results

### The InDelphi mESC-trained model accurately predicts the gene editing outcomes of CRISPR/Cas9 in *Xenopus tropicalis* and outperforms other tested prediction models

**Figure 1:**
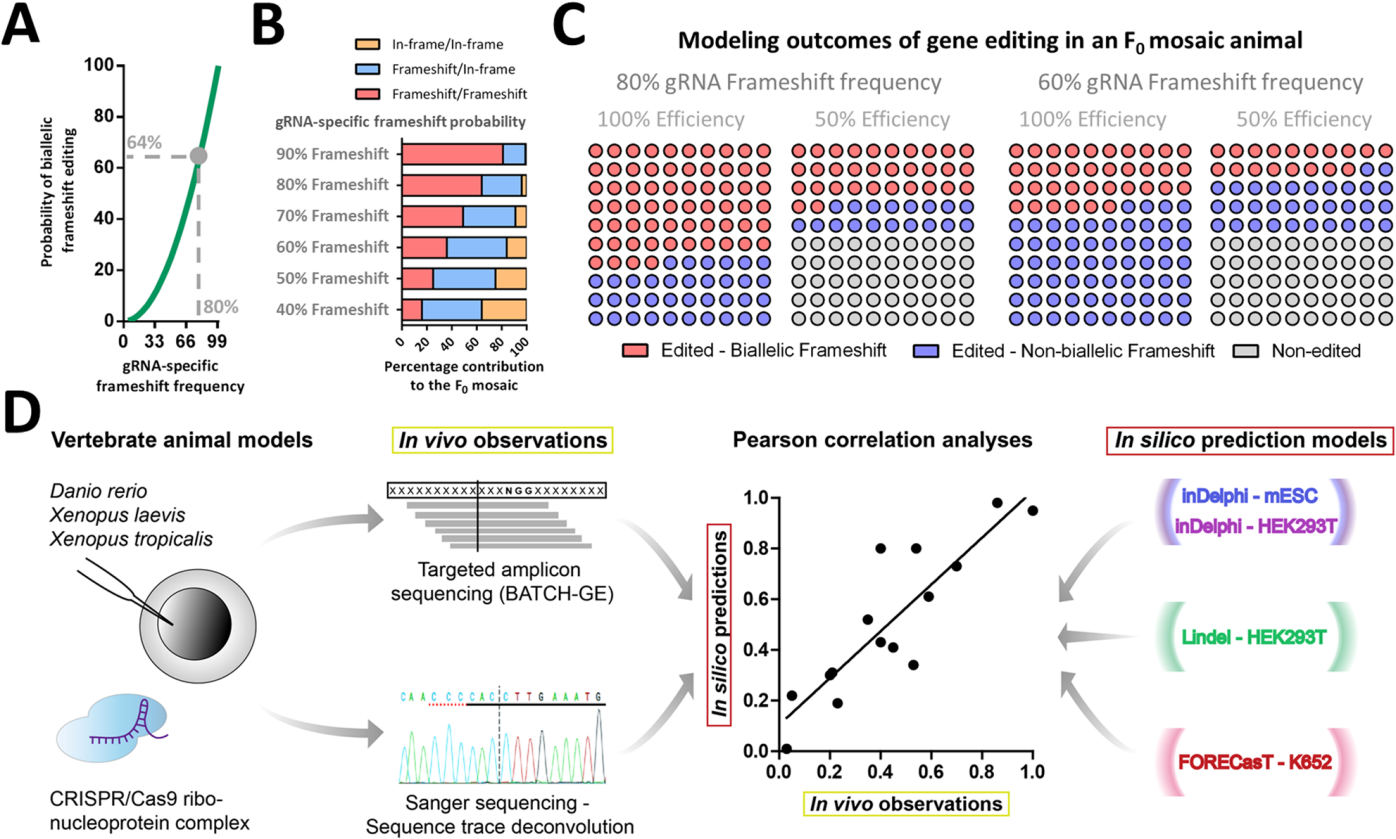
Theoretical models of how gRNA-specific efficiencies and frameshift gene editing outcome probabilities influence the cellular composition and percentage of protein knockout cells in a mosaic F_0_ animal model. (**A**) There is a non-linear relationship between gRNA-specific probability of obtaining a frameshift gene editing outcome (x-axis) and the probability of obtaining a biallelic frameshift gene editing outcome in a single cell (y-axis). *E.g.* upon a gRNA-specific frameshift frequency of 80%, the probability of a single biallelic edited cells to be biallelic frameshift mutant is 64% (0.80*0.80). (Grey demarcation). (**B**) Examples of theoretical outcomes of gene editing (presuming 100% on-target efficiency) in an F_0_ mosaic varying one parameter: gRNA-specific probability of frameshift editing. (**C**) Examples of theoretical outcomes of gene editing in an F_0_ mosaic varying two parameters: gRNA-specific probability of frameshift editing and gRNA-specific on-target efficiency. *E.g.* for a 100% efficient gRNA with an 80% gRNA-specific probability of frameshift editing, we expect 64% of the cells to be biallelic frameshift mutant (see grey demarcation in **A**). Please note, blue circles represent cells that are biallelic gene edited, but retain at least one in-frame mutation and cannot be considered complete protein knock-out. (**D**) Flowchart representing the pipe-line for investigating the correlations between experimentally observed in vivo gene editing outcomes and gene editing outcomes projected by computational prediction models.

In *X. tropicalis*, where CRISPR/Cas9 reagents are typically injected as a Cas9/gRNA-ribonucleoprotein complex at an early developmental stage (2 to 16 cell stage), rapid embryonic cell division will generate a spectrum of mosaic CRISPR/Cas9 mutations, with dissimilar DSB repair events in different cells of the animal. This provides a unique opportunity to gain insight into how CRISPR/Cas9 DSBs are repaired in these vertebrate embryos. The ratios of cells within the mosaic presenting with certain INDEL variants is representative of the probabilistic outcomes of gene editing towards that specific mutation. In fact, there is a non-linear relation between the on-target gRNA ratio of frameshift editing and the percentage of biallelic mutant cells within the mosaic (Fig. 1A,B). When gRNA on-target efficiency reduces, this effect further inflates, leading to an ever-increasing contribution of cells in the developing embryo that still express functional protein of the gene under scrutiny (Fig. 1C).

**Figure 2:**
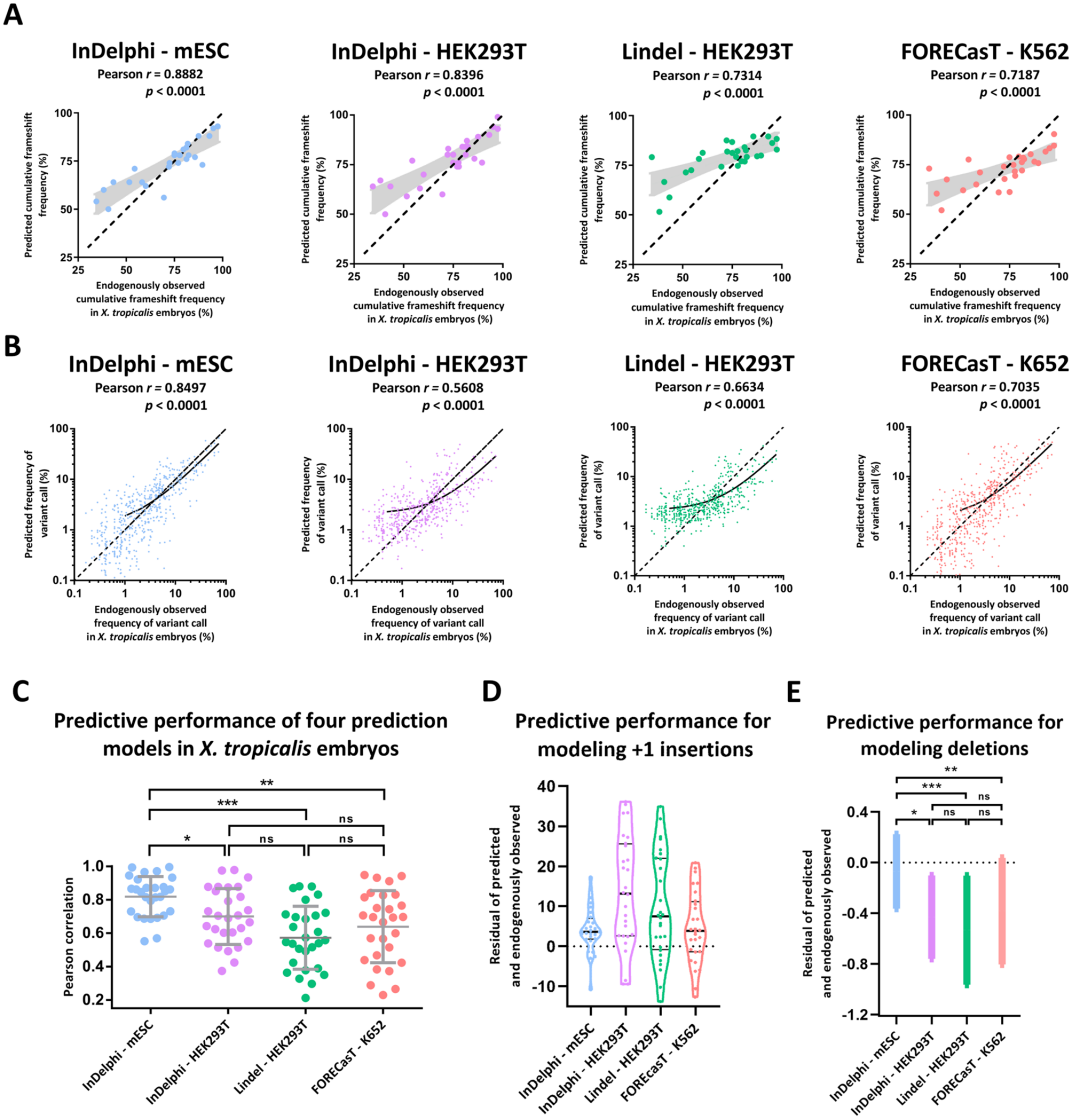
The InDelphi prediction model, trained in mESC cells, accurately predicts CRISPR/Cas9 gene editing outcomes and outperforms several other prediction models in *X. tropicalis* embryos. (**A**) Scatter plot with model-predicted cumulative frameshift gene editing frequencies correlated to experimentally observed cumulative frameshift gene editing frequencies, for each sgRNA (*n* = 28) separately, in *X. tropicalis* embryos. Black demarcated lines show the perfect correlation *r* = *1*. Light-grey shows the standard error of the best-fit linear regression line. (**B**) Scatter plot with model-predicted INDEL patterns correlated to experimentally observed INDEL patterns, for all gRNAs simultaneously. Black lines show linear regression models of all correlations. Black demarcated lines show the perfect correlation *r* = *1.* (**C**) Correlations between model-predicted and experimentally observed INDEL patterns, for each gRNA separately. Error bars represent mean ± SD. (***p < 0.001; **p < 0.01; *p < 0.05; ns = not significant; Shapiro–Wilk (*p* > 0.05); Levene (*p* < 0.05); One-way Welsh ANOVA to adjust for unequal variances (*p* < 0.001), with Games-Howell multiple comparisons) (Table S2). (**D**) Violin plots of the residuals (predicted frequency—observed frequency) between model-predicted and experimentally observed frequency of + 1 insertion gene editing outcome. (**E**) The SEM of the mean residual difference (predicted frequency—observed frequency) between model-predicted and experimentally observed frequency of all deletion variants modeled.

We had previously observed significant correlation between experimental *ezh2* CRISPR/Cas9 gene editing outcomes in *X. tropicalis* embryos, and the predictions obtained for the InDelphi model trained in mouse embryonic stem cell modus (further abbreviated as: InDelphi-mESC) (Fig. S1)^31^. We next questioned whether this observation would hold true in a larger dataset and also for other recently reported prediction models. For this, we analyzed the gene editing outcomes of 28 different gRNAs to 21 distinct genes injected individually as Cas9/gRNA-ribonucleoproteins in *X. tropicalis* embryos. We employed targeted amplicon sequencing of the CRISPR/Cas9 target sites to obtain experimental DSB repair outcomes at high-resolution in the injected embryos^32,33^. This permitted us to compare in vivo gene editing outcomes in *X. tropicalis* embryos to the in silico predictions of four previously trained models: InDelphi-mESC, InDelphi adapted to HEK293T cellular context (further abbreviated as: InDelphi-HEK293T), Lindel (trained in HEK293T cells) and FORECasT (trained in K562 cells) (Fig. 1D)^27–29^. Please note that both InDelphi modules (mESC and HEK293T) make identical cell-type-independent predictions for deletions, while only using cell-type-specific data to predict the ratio of 1-bp insertions to deletions^27^. First, we performed correlation analysis between model-predicted and experimentally observed cumulative frameshift gene editing frequencies, for each sgRNA separately (*n* = 28). Here, InDelphi-mESC demonstrated superior performance (*r* = 0.89, *p* < 0.0001), when compared to the other prediction models: InDelphi-HEK293T (*r* = 0.84, *p* < 0.0001), Lindel (*r* = 0.73, *p* < 0.0001) and FORECasT (*r* = 0.72, *p* < 0.0001) (Fig. 2A). Second, we performed correlation analysis between model predicted INDEL patterns versus experimentally observed frequencies of INDEL variants, for all gRNAs simultaneously. This revealed, in line with previous, superior predictive performance of InDelphi-mESC (*r* = 0.85, *p* < 0.0001), when compared to the other prediction models: InDelphi-HEK293T (*r* = 0.56, *p* < 0.0001), Lindel (*r* = 0.66, *p* < 0.0001) and FORECasT (*r* = 0.70, *p* < 0.0001) (Fig. 2B). Of note, lower frequency gene editing outcomes are predominantly mispredicted in HEK293T-specific predictions models.

Third, we analyzed the correlations between model predicted INDEL patterns to experimentally observed frequencies of INDEL variants, for each gRNA separately. We show that InDelphi-mESC is able to accurately predict gene editing outcomes, for a single gRNA, in *X. tropicalis* embryos with an average Pearson correlation coefficient of 0.82 ± 0.12 (Fig. 2C; Fig. S2). As such, InDelphi-mESC outperforms InDelphi-HEK293T (*r* = 0.70 ± 0.17), Lindel (*r* = 0.57 ± 0.19) and FORECasT (*r* = 0.64 ± 0.22) Pearson correlation coefficients. Interestingly, the comparison of model-predicted and experimentally observed mutations, revealed that HEK293T-specific predictions consistently overestimate the frequency of + 1 insertions when compared to experimental editing outcomes in *X. tropicalis* embryos (Fig. 2D). Thus + 1 insertions appear to be a major editing outcome in HEK293T cells but less so in *X. tropicalis* embryos and mESC cells, which is reflected in their respective correlations (Fig. 2B,C). In line, the residuals between model-predicted and experimentally observed frequency of all deletion outcomes of gene editing, revealed that InDelphi also provides superior modeling of deletion outcomes compared to Lindel and FORECasT (Fig. 2E). Taken together, we show that the InDelphi-mESC model is capable of accurately predicting CRISPR/Cas9 gene editing outcomes occurring in vivo in early developing *X. tropicalis* embryos, upon micro-injection of CRISPR/Cas9 ribonucleoprotein complexes. InDelphi-mESC significantly outperforms predictive models trained in more differentiated cell lines.

### Accurate correlations between gene editing outcomes predicted by InDelphi-mESC and in vivo gene editing outcomes determined by Sanger sequencing and sequence trace deconvolution in two species of *Xenopus*

**Figure 3:**
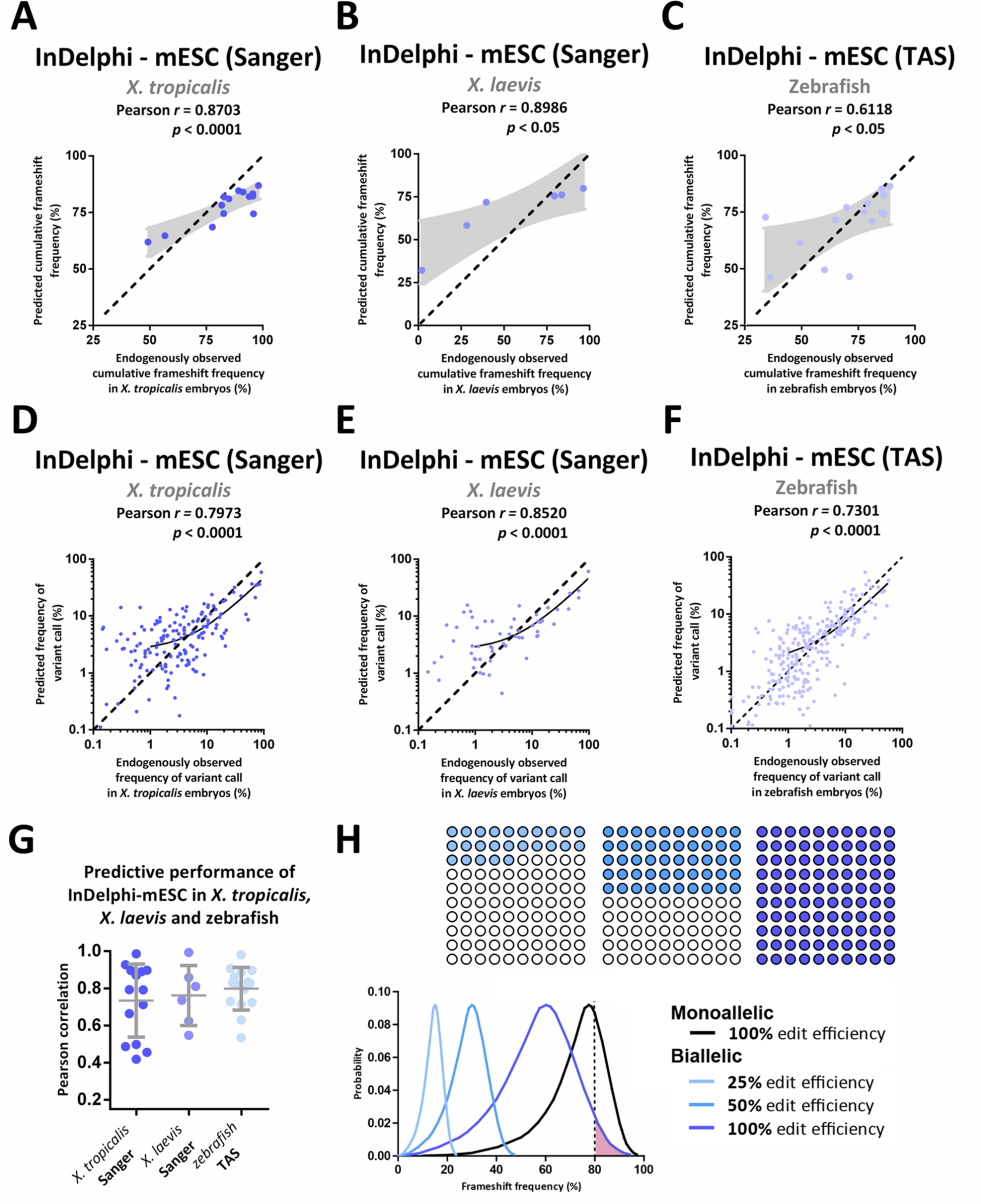
The InDelphi-mESC model accurately predicts CRISPR/Cas9 gene editing outcomes in *X. tropicalis, X. laevis* and zebrafish embryos which can be exploited to identify high-frameshift frequency gRNAs. (**A**–**F**) Scatter plot with InDelphi-mESC-predicted cumulative frameshift gene editing frequencies correlated to experimentally observed cumulative frameshift gene editing frequencies, for each sgRNA separately, in *X. tropicalis* (*n* = 14) (Panel **A**), in *X. laevis* (*n* = 6) (Panel **B**) and in zebrafish (*n* = 15) embryos (Panel **C**). Scatter plot with InDelphi-mESC-predicted INDEL patterns correlated to experimentally observed INDEL patterns, for all gRNAs simultaneous, in *X. tropicalis* (*n* = 14) (Panel **D**), in *X. laevis* (*n* = 6) (Panel **E**) and zebrafish (*n* = 15) (Panel **F**) embryos. Black demarcated lines show the perfect correlation *r* = *1*. Light-grey areas show the standard error on the best-fit linear regression line. Black lines show linear regression model. (**G**) Correlations between model-predicted INDEL patterns to experimentally observed INDEL patterns, for each gRNA separately. Correlations for *X. tropicalis* embryos (*n* = 14) (dark blue) and *X. laevis* embryos (n = 6) (middle blue) analyzed by Sanger sequencing and sequence trace decomposition. Correlations for zebrafish embryos analyzed by targeted amplicon sequencing (TAS) (*n* = 15) (light blue). (**H**) Using the distribution of the expected probability of frameshift frequency for a large dataset of SpCas9 human target sites in mESC cells from Shen et al. 2018 (black line—monoallelic)^27^, we draw the derivative distribution of the probability of a randomly designed gRNA to generate biallelic frameshift editing. This distribution is shown for different editing efficiencies within the F_0_ mosaic animal: 100%, 50% and 25% (in reducing intensities of blue—100 circles, each circle represents a cell within a total mosaic of a 100 cells). *E.g.* The probability of a randomly designed gRNA to yield more than 80% biallelic frameshift mutant cells in a developing mosaic, assuming 100% efficiency, is the area under curve highlighted in pink and represents only a 3.24% probability.

To validate and extend our observations beyond a possible laboratory-dependent bias for gRNA selection and by using alternatives for targeted amplicon sequencing approaches, we compared InDelphi-mESC predictions to experimental gene editing outcomes in *X. tropicalis* (*n* = 14) and *X. laevis* (*n* = 10), determined by Sanger sequencing and sequence deconvolution analysis with the Inference of CRISPR Edits (ICE) algorithm^34^. In *X. tropicalis* embryos, InDelphi-mESC was also able to accurately predict the experimental cumulative frequency of frameshift editing, for each gRNA separately (*r* = 0.87; *p* < 0.0001) (Fig. 3A), as well as INDEL patterns across all gRNAs simultaneously (*r* = 0.80*; p* < 0.0001) (Fig. 3D). Further, InDelphi-mESC-predicted INDEL patterns correlate with the frequencies of experimentally observed INDEL patterns in the developing *X. tropicalis* embryo, for each gRNA separately (*r* = 0.73 ± 0.20) (Fig. 3G, Fig. S3).

Next, we tested whether these observations could be expanded to *Xenopus laevis* embryos. Using an identical injection and Sanger sequence deconvolution approach, we observed that 60% (*n* = 10) of gRNAs showed significant (*p* < 0.05) Pearson correlations (*r* = 0.76 ± 0.16) between InDelphi-mESC-predicted and experimentally observed INDEL patterns (Fig. 3G; Fig. S4). We speculate that the lack of correlation for the remaining four gRNAs in *X. laevis* is the consequence of underpowered in vivo observations of the heterogeneous outcomes of gene editing. Namely, in the slower developing *X. laevis* embryos, it can be rationalized that gene editing will occur at an earlier developmental stage than is the case for *X. tropicalis* and this will lead to a lower number of discrete DSB repair events and thereby a lower degree of mosaicism. This interferes with the premise that the mosaicity of the early embryo is representative to the probabilistic outcomes of gene editing towards a specific variant. Despite these limitations, gRNAs with sufficiently-powered experimental gene editing outcome observations (n = 6), featured accurate prediction of experimental cumulative frequency of frameshift editing, for each gRNA separately (*r* = 0.90; *p* < 0.05) (Fig. 3B), and INDEL patterns across all gRNAs simultaneously (*r* = 0.85*; p* < 0.0001) by InDelphi-mESC (Fig. 3E).

Taken together we show that, in some cases (n = 4) Sanger sequencing and sequence trace deconvolution on a limited subset of embryos, can lack power for quantitatively assessing heterogeneous gene editing outcomes occurring in vivo. Despite this limitation, we demonstrate significant correlations between InDelphi-mESC model predicted gene editing outcomes and experimental editing outcomes for 83% (n = 24) of gRNAs across both *X. laevis* and *X. tropicalis* using Sanger sequencing and trace deconvolution.

### InDelphi mESC-trained model accurately predicts the gene editing outcomes of CRISPR/Cas9 in zebrafish and allows for rational gRNA design to favor high frameshift frequency gene editing outcomes

Finally, we investigated whether InDelphi-mESC can also predict gene editing outcomes in the evolutionary more distant teleost phylogenetic lineage. For this, we compared InDelphi-mESC model predictions to in vivo gene editing outcomes in zebrafish embryos, identified by targeted amplicon sequencing^32^, upon micro-injection of CRISPR/Cas9 ribonucleoproteins (*n* = 15). Once again, we demonstrate that InDelphi-mESC is able to accurately predict cumulative frameshift frequencies, for each gRNA separately (*r* = 0.62; *p* < 0.05) (Fig. 3C), and INDEL patterns across all gRNAs simultaneously (*r* = 0.73; *p* < 0.001) (Fig. 3F). In line, the INDEL pattern gene editing outcomes, for each gRNA separately, are also predictable by the InDelphi-mESC model (*r* = 0.80 ± 0.12) (Fig. 3G, Fig. S5).

### Integrating CRISPRscan and the InDelphi-mESC model allows identification of efficient high frameshift frequency gRNAs in *X. tropicalis*

**Figure 4:**
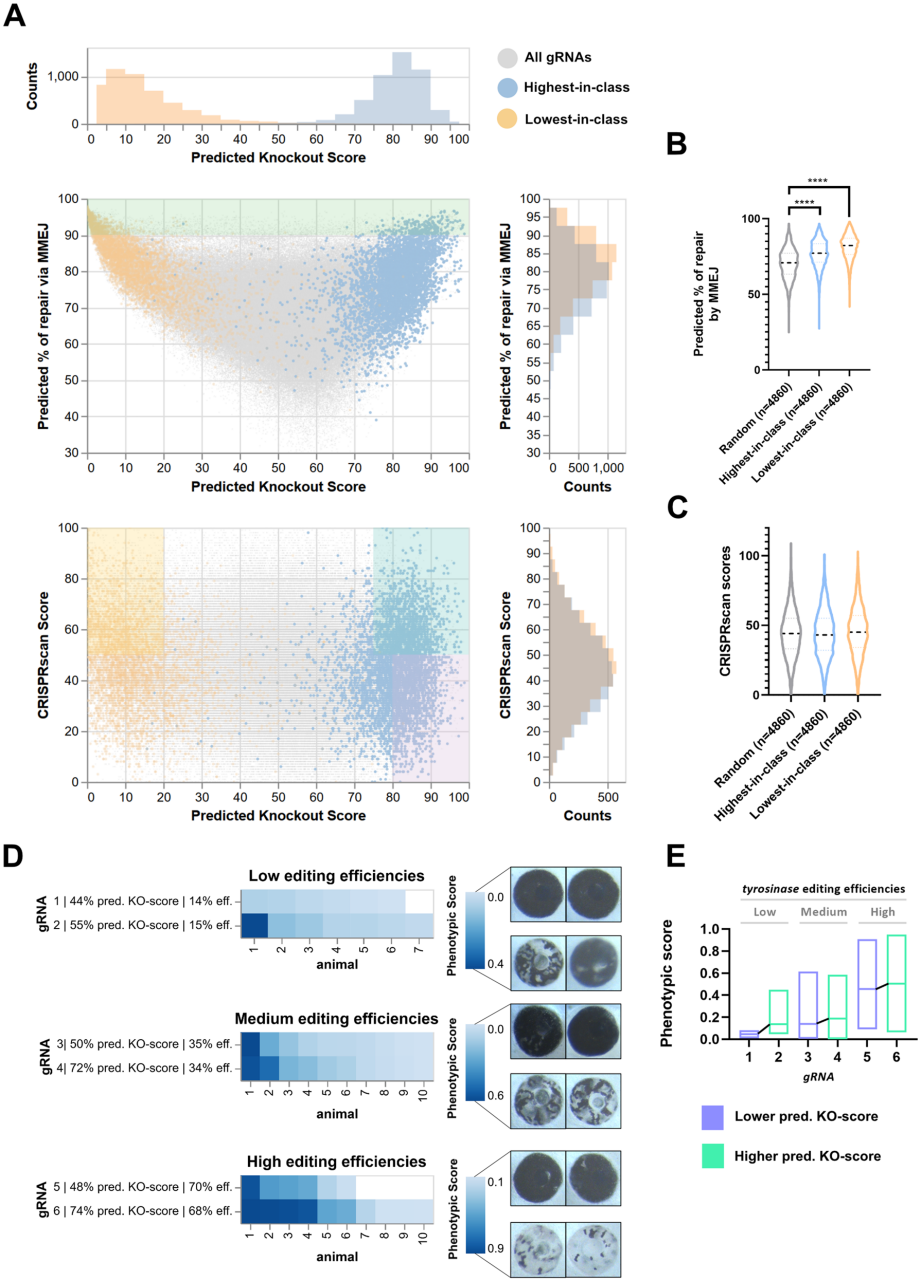
Integrating CRISPRscan and the InDelphi-mESC model allows identification of efficient high frameshift frequency gRNAs in *X. tropicalis.* (**A**) Scatterplot with marginal histograms demonstrating for 339,693 gRNAs across the coding sequence for 4,860 *X. tropicalis* genes the relationships between calculated CRISPRscan score, InDelphi-mESC predicted frequency of MMEJ repair and InDelphi-mESC predicted knockout-score (KO-score). KO-score is defined as the predicted percentage of cells with biallelic out-of-frame mutations within the pool of all mutant cells (*i.e.* in-frame and out-of-frame; mono- and bi-allelic) in the mosaic mutant embryo and is calculated as the square of the frameshift frequency predicted by InDelphi-mESC. For each gene (*n* = 4,860), the gRNA with the highest predicted KO-score (Highest-in-class) is highlighted in blue, while the gRNA with the lowest predicted KO-score (Lowest-in-class) is highlighted in orange. Demarcations illustrate those quadrants where gRNAs suffice to certain cutoff thresholds. Ideally, designed gRNAs fall within the aquamarine demarcation (high predicted KO-score, high CRISPRscan score), but not the orange (low predicted KO-score, high CRISPRscan score) or purple demarcation (high predicted KO-score, low predicted CRISPRscan score). (**B**) Violin plot illustrating that highest-in-class gRNAs and lowest-in-class gRNAs have a higher predicted percentage of repair by microhomology-mediated end joining than a random selection of guides. (****p < 0.001—Table S2). (**C**) No distinct difference in calculated CRISPRscan scores between highest-in-class gRNAs, lowest-in-class gRNAs and a random selection of gRNAs. (**D**) Comparison of three pairs of gRNAs targeting the second exon of the *tyrosinase* gene responsible for pigmentation in *X. tropicalis*. As these three pairs of guides have very similar genome editing efficiencies, as determined by targeted amplicon sequencing, the impact of differential predicted KO-scores on phenotypic penetrance is revealed. (**D**, **E**) Phenotypic scoring is based on retinal pigmentation at Nieuwkoop-Faber stage 38 and a trend is observed where guides with higher predicted KO-scores yield a higher phenotypic score under very similar genome editing efficiencies.

In order to investigate the possible application of InDelphi (together with CRISPRscan) for efficient gRNA design in future experimental settings, we calculated CRISPRscan scores, InDelphi-mESC predicted frequency of MMEJ repair and InDelphi-mESC predicted knockout-score (KO-score) for 339,693 gRNAs across the coding sequence for 4,860 *X. tropicalis* genes. The KO-score is defined as the predicted percentage of cells with biallelic out-of-frame mutations within the pool of all mutant cells (*i.e.* in-frame and out-of-frame; mono- and bi-allelic) in the mosaic mutant embryo and is calculated as the square of the frameshift frequency predicted by InDelphi-mESC (Fig. 4A). We identified for each of these individual genes: the guide RNA with the highest predicted KO-score (annotated as ‘highest-in-class’, Fig. 4A—blue), the lowest predicted KO-score (annotated as ‘lowest-in-class’, Fig. 4A—orange), on a background of all possible gRNAs targeting these genes (Fig. 4A—grey). When comparing the highest-in-class and lowest-in-class gRNA for each gene to a random sample of 4,860 gRNAs, we observe enrichment towards an increased repair by microhomology-mediated mechanisms (p < 0.0001) (Fig. 4B). Nevertheless, only 5.3% of highest-in-class gRNAs exceed a threshold of over 90% repair by MMEJ (Fig. 4A—green demarcated). This indicates that only considering local MMEJ strength as a factor for maximizing phenotypic penetrance could be insufficient as frameshifting mutations can also be enriched for gRNAs producing heterogeneous editing outcomes. Next, we investigated the CRISPRscan scores as a measure for predicted in vivo editing efficiencies for these highest-in-class guides, expected to be the best candidates for maximizing phenotypic penetrance. This revealed that, as expected, highest-in-class, lowest-in-class and a random selection of 4,860 gRNAs have similar CRISPRscan score distributions (Fig. 4C). In practice, merely considering CRISPRscan score during gRNA design could be insufficient to obtain a high KO phenotype. In fact, we observe that gRNAs scoring well on CRISPRscan (score > 50) can have a low predicted KO-score and some can even be characterized as lowest-in-class for that gene (Fig. 4A, orange demarcated). Conversely, only considering KO-score could be similarly insufficient as these gRNAs could have a low CRISPRscan score, yielding lack of in vivo effectiveness (Fig. 4A, purple demarcated). In conclusion, we determine that 30% of the screened genes (n = 4,860) have a highest-in-class gRNA with a KO-score exceeding 75 and a CRISPRscan score exceeding 50, a score considered sufficient for efficient in vivo editing (Fig. 4A, aquamarine demarcated). For the remaining genes, guides can be identified with a slightly lower predicted KO-score, but with CRISPRscan scores exceeding this threshold. It is worth noting that sacrificing some of the CRISPRscan score in order to obtain a gRNA with a higher predicted KO-score is beneficial, as cutting efficiency can be improved in an experimental setup by increasing the injected dose of CRISPR/Cas9 RNP. In contrast, the frameshift frequency pattern of the chosen gRNA is inherent to its sequence context and cannot be altered experimentally.

In order to relate phenotype penetrance variability to different predicted KO-scores, we targeted the second exon of the *tyrosinase (tyr)* gene using six distinct gRNAs. For this, each *tyr* gRNA respectively was coinjected with Cas9 recombinant protein in both blastomeres of two-cell stage *X. tropicalis* embryos. Absence of retinal pigmentation was used as a proxy for the efficiency of *tyr* biallelic frameshift editing. Targeted amplicon sequencing revealed very similar genome editing efficiencies between *tyr*^gRNA1^ and *tyr*^gRNA2^ (*low*—around 14%), *tyr*^gRNA3^ and *tyr*^gRNA4^ (*medium*—around 34%), *tyr*^gRNA5^ and *tyr*^gRNA6^ (*high*—around 68%) (Fig. 4C, Table S3). The similar gene editing efficiencies within these three pairs allows directly measuring the impact of the predicted KO-scores on phenotypic penetrance. Quantification of residual eye pigmentation at Nieuwkoop-Faber stage 38 revealed a clear trend where guides with higher predicted KO-scores consistently yielded a higher phenotypic score under very similar genome editing efficiencies (Fig. 4D-E, Fig. S6). This in vivo functional experiment strengthens our notion that selecting a gRNA with a predicted high frameshift frequency using the InDelphi-mESC model can help maximize CRISPR/Cas9 phenotypic penetrance.

## Discussion

In this manuscript we investigated the power of three established computational prediction modules for anticipating template-free CRISPR/Cas9 editing outcomes in three vertebrate species commonly used as biomedical model organisms. We show the InDelphi neural network mESC module to accurately forecast CRISPR/Cas9 editing outcomes in embryos of *Xenopus* and *Danio rerio*. Our observations strengthen the developing paradigm that double-strand break repair after template-free CRISPR/Cas9 editing is predictable, but may proceed in a cell-type specific manner. We hypothesize that the undifferentiated pluripotent state of mESC cells, wherein InDelphi-mESC was trained, most closely resembles the epigenetic, transcriptomic and post-transcriptional context of an early developing pluripotent vertebrate embryo. This directly impacts rational design of CRISPR/Cas9 experiments, implementing in the design process selection of gRNAs that favor frameshift gene editing outcomes, thereby improving F_0_ knockout studies. Ideally, the InDelphi-mESC model is exploited to identify “precise” gRNAs with inherently precise editing outcomes, defined as homogeneous DNA repair outcomes and enrichment for specific frameshift variants. Namely, our data shows that the InDelphi-mESC model is able to accurately predict in vivo CRISPR/Cas9 gene editing outcomes, only based on local sequence context, in early embryos of different vertebrate species commonly used as model organisms.

We suggest using the InDelphi-mESC model to cherry-pick gRNAs predicted to yield high rates of frameshift gene editing outcomes, allowing maximization of protein knockout in vertebrate F_0_ mosaic CRISPR/Cas9 edited animals and thereby increasing phenotypic penetrance. When InDelphi-mESC is employed to predict frameshift frequencies occurring at 13,273,449 SpCas9 human target sites after CRISPR/Cas9 editing^27^, mathematical modeling reveals that a randomly designed gRNA, with assumed full on-target efficiency, only has a 3.24% theoretical probability of resulting in equal or larger than 80% of the cells in the F_0_ mosaic being biallelic frameshift mutations (Fig. 3H)^27^. As most gRNAs will not reach full on-target efficiency, this effect will become even more pronounced in an in vivo setting. This clearly illustrates the advantage of selecting gRNAs with high frameshift prediction for exposing phenotypes in F_0_ mosaic animals. In addition, when breeding F_0_ animals generated using precise gRNAs, a major contribution of predominant gene editing outcomes to the germline can be expected. Implementation of InDelphi-mESC in the gRNA design process should facilitate the generation of frameshift heterozygous mutant F_1_ animals upon out breeding, and potentially accelerate the creation of homozygous mutant F_1_ animals by directly breeding F_0_ founders. In addition, genome editing outcome predictions are useful to determine both positive and negative selection pressure on cellular fitness occurring as a consequence of underlying biological processes, such as—but not limited to—tumorigenesis and cancer^31,35^.

Recently, for gene knockout studies, the use of frameshift-inducing gRNAs that cause nonsense-mediated decay (NMD) of the transcript has been contested. Using experiments in zebrafish and mouse embryonic stem cells, it was found that some CRISPR mutated transcripts that are subjected to NMD are able to induce transcriptional adaptation and compensation resulting in a less severe phenotype^36^. Hence, complete deletion of the targeted gene by deploying flanking gRNAs has been advocated. However, the underlying mechanism, while being clearly sequence-determined, still is poorly defined. Hence, at the moment it remains to be seen how universal this phenomenon is. Additionally, a major concern for deleting large or complete parts of a particular gene is the concomitant removal of possible intronic non-coding regulatory elements and RNAs, which can add to off-target concerns already inherent to the CRISPR/Cas9 system.

Evidently, predictions of gene editing outcomes can also be harnessed to specifically design gRNAs with known dominant or loss-of-function in-frame mutations. This can be exploited for structure–function studies of protein variants in a cell or development biology context^19^. Obviously, the analysis of such experiments would be challenging in the F_0_ generation but achieving dominant editing of specific in-frame variants heavily increases the chance of passing the exact genotype of interest to the F_1_ generation. Furthermore, to deal with the possible NMD-induced transcriptional adaptation and compensation mentioned above, it was recently argued that the use of gRNAs selecting for in-frame mutations in domains essential for protein function could be another approach to produce loss-of-function alleles^37^. In this scope, InDelphi could similarly be employed to identify gRNAs with high predicted in-frame frequencies (our lowest-in-class gRNAs). In conclusion, we show that the InDelphi-mESC model allows for the rational design of gRNAs that have high efficiency CRISPR/Cas9-mediated loss-of-function INDEL mutations in vertebrate developing embryos, allowing to maximize F_0_ and F_1_ phenotype penetrance. Evidently gRNA design should also integrate prediction of cutting efficiency (*e.g.* via CRISPRscan). We believe these findings to have direct implications for *Xenopus* and zebrafish CRISPR/Cas9 genome engineering and should be included in the standard guide RNA design process.

## Material and methods

### Guide RNA synthesis and microinjection

For the *Xenopus tropicalis* experiments, gRNAs were designed with the CRISPRscan software package^15^, generated and quality controlled as previously described^23^, employing the oligos shown in supplementary table S1A. When using AltR-crRNA from Integrated DNA Technologies (IDT, USA), crRNA was annealed with AltR-tracrRNA prior to injections according to the guidelines of the company. Recombinant Cas9 protein was commercially obtained (PNA Biosciences) or in-house generated as previously described^38^. *Xenopus tropicalis* embryos were microinjected in either the one-, two- or four-cell stage with injection mixes containing precomplexed gRNA (500–750 pg) and Cas9 protein (1 ng). Embryos were lysed for 2 h up to overnight in lysis buffer (50 mM Tris pH 8.8, 1 mM EDTA, 0.5% Tween-20, 2 mg/ml Proteinase K) and genotyped by PCR amplification using primers indicated in supplementary table S1A. In the *Xenopus laevis* experiments the gRNAs were designed with the CRISPRscan (https://www.crisprscan.org) or CRISPRdirect (https://crispr.dbcls.jp) software package (Table S1B)^15,39^. These were secondarily verified for no or low potential of off-targets with GGGenome (https://gggenome.dbcls.hp/) using the most up to date version of the *X. laevis* genome available at the time of gRNA design. Recombinant NLS-Cas9-NLS protein was purified and the sgRNAs synthesized in the Horb lab. Embryos were injected at one cell stage with 10 nl of a mix containing 0.1% of TexasRed dextran (40,000 MW, neutral) (Invitrogen, Eugene, OR), 1.5 ng Cas9 protein and 0.75 ng of sgRNA. One- or two-days post injection genomic DNA was extracted from whole embryos (GenElute Mammalian Genomic DNA Miniprep Kit, Sigma-Aldrich, St. Louis, MO), the target regions were PCR amplified (Taq DNA Polymerase, New England Biolabs, Ipswich, MA) using primers indicated in supplementary table S1B, the PCR products were cleaned (EconoSpin Spin Column For DNA, Epoch Life Science, Missouri City, TX) and sent for Sanger sequencing (GENEWIZ, South Plainfield, NJ). For the zebrafish experiments, single-guide RNA (sgRNA) molecules were designed with the CRISPRdirect software (https://crispr.dbcls.jp/)36, and were produced as previously reported^31^. Briefly, in vitro transcription (MEGAshortscript T7 Transcription Kit, Invitrogen, cat. nr. AM1354) was carried out on target-specific double-stranded DNA molecules (gBlocks), followed by RNA purification (MEGAclear Kit, Life Technologies, cat. nr. AM1908) and quantity and integrity assessment. One-cell stage zebrafish embryos were injected with CRISPR/Cas9 components in the cell, as previously described^31^. At 1-day post fertilization (dpf), DNA extraction was performed on a pool of 20 embryos (KAPA Express Extract DNA Extraction Kit, Kapa Biosystems, KK7103). The resulting DNA was stored at -20 °C for subsequent targeted PCR amplification and deep sequencing using primers indicated in supplementary Table S1C. For quantification of loss of eye pigmentation in *tyrosinase* RNP injected *X. tropicalis*, pictures where acquired with a Carl Zeiss StereoLUMAR.V12 stereomicroscope equipped with an AxioCam MRc using Zen Blue and processed as follows for quantification. Pictures were cropped maintaining all voxel ratios to 365 × 365 pixels to contain only the eye. Using thresholding we masked the pigmented part of the eye for quantification using following FIJI macro: [run("8-bit"); setThreshold(1, 30); setOption("BlackBackground", true); run("Convert to Mask"); run("Analyze Particles…", "display summarize");]. Quantification of masked pixels generated values between 4,011 and 79,408. All measurements were normalized to the most pigmented eye using following formula: [(79,408-masked_pixels)/79,408]. For genotyping of *tyr* mutants, single embryos where lysed and processed for targeted amplicon sequencing identical as described above.

### Comparison of in vivo CRISPR/Cas9 editing outcomes to in silico prediction models

All targeted amplicon sequencing was performed and analyzed as previously described^33^. For InDelphi modeling (https://InDelphi.giffordlab.mit.edu/)^27^, sequence context input was 80 bp (40 bp upstream and 40 bp downstream of the cleavage site) in both mESC and HEK293T input mode. For correlation between InDelphi predictions and in vivo observations, wild-type reads and reads containing insertions > 1 were omitted from the experimental sequencing results to allow comparison to InDelphi predictive modeling software output. Residual reads were normalized to 100% to obtain experimentally observed frequencies for each retained mutant read variant, which were correlated to in vivo observations using Pearson correlation. For FORECasT and Lindel modeling, sequence context input was 65 bp (30 bp upstream and 35 bp downstream of the cleavage site) and ran respectively in the GUI (https://partslab.sanger.ac.uk/FORECasT) or in the command line of Python3 using GitHub-deposited code (https://github.com/shendurelab/Lindel)^29,40^. To allow direct comparison of the predictive strengths of these different models, we curated all predictions to be in-line with the editing outcome classes of the InDelphi model, thus comparing the frequencies of gene editing outcomes containing insertions + 1 bp up to the deletion that exceeds the combined 99% cumulative probability of all events using InDelphi in mESC mode. This approach disfavors disproportionate weighting of low-frequency large deletion variants in the Pearson correlation. All correlations were performed using Pearson correlation imbedded in the GraphPad Prism software package. For Sanger sequencing and trace deconvolution we employed the ICE algorithm (Synthego—available from: https://ice.synthego.com/)^34^. Raw Sanger data was implemented in the ICE algorithm for both the wild type control as well as the injected samples along with the applicable gRNA sequence. For every gRNA, generated ICE data from at least three different injected embryos was averaged out and frequencies were normalized (as such containing all 1 bp insertions and up to 20 bp deletions) and correlated to the InDelphi predictions (mESC input mode). Statistical analysis in relation to Fig. 2C performed with SPPS software version 26 (IBM).

### Identifying gRNAs in 4,860 *X. tropicalis* genes and predicting in vivo editing outcomes and CRISPRscan scores

InDelphi-mESC pretrained model was obtained (https://github.com/maxwshen/inDelphi-model) and deployed using scikit-learn v0.21.3. CRISPRscan sgRNA activity regression model was implemented as described in Moreno-Mateos et al.^15^. Using a custom Python3 pipeline, guide RNAs were designed across the first 4,860 entries of the coding sequences (CDS) of the *X. tropicalis* v9.1 genome assembly obtained from Xenbase (https://ftp.xenbase.org/pub/Genomics/JGI/Xentr9.1/sequences/XENTR_9.1_Xenbase.cds.fa)^41^. For each of the 4,860 entries gRNAs compatible with T7 transcription were retained, scored with CRISPRscan, analyzed using the InDelphi mESC model to determine frameshift frequency and the percentage of repair by MMEJ. Knockout-score (KO-score) is defined as the square of the InDelphi-mESC predicted frameshift frequency. For each gene the gRNA with the highest predicted KO-score (highest-in-class) and the lowest predicted KO-score (lowest-in-class) was determined. Further data analysis to obtain percentages of gRNAs accommodating certain CRISPRscan or KO-score thresholds was performed using Pandas v0.25.1. All data was visualized using the Altair v4.0.1 package.

### Ethical statement

Experiments in *Xenopus tropicalis* were performed using guidelines approved by the CCHMC Institutional Animal Care and Use Committee (IACUC2019-0,053) and the Ethical Committee for Animal Experimentation, Ghent University, Faculty of Science and VIB-Site Ghent (EC2018-079). All animal experiments with *Xenopus* performed at the National Xenopus Resource were approved by the MBL Institutional Animal Care and Use Committee (19–03). Approval for zebrafish experiments was provided by the local committee on the Ethics of Animal Experiments (Ghent University Hospital, Ghent, Belgium). Experiments in zebrafish were performed according to the guidelines outlined in Permit Number: ECD 14/31 and ECD 17/41.

## Supplementary information

Supplementary file1

Supplementary file2

Supplementary file3

Supplementary file4
