# Supplementary material for: Maximizing CRISPR/Cas9 phenotype penetrance applying predictive modeling of editing outcomes in Xenopus and zebrafish embryos
Source: Sci Rep. 2020 Sep 4;10:14662. doi: 10.1038/s41598-020-71412-0 (PMC7473854; doi:10.1038/s41598-020-71412-0)
Supplement: Supplementary file 1 — Supplementary file1 [file 41598_2020_71412_MOESM1_ESM.pdf]

Supplementary Figures to:

Maximizing CRISPR/Cas9 phenotype penetrance applying predictive modeling of editing outcomes in *Xenopus* and zebrafish embryos

Thomas Naert[1,2,6], Dieter Tulkens[1,2,6], Nicole A. Edwards[3], Marjolein Carron[1,4], Nikko-Ideen Shaidani[5], Marcin Wlizla[5], Annekatrien Boel[4], Suzan Demuynck[1,2], Marko E. Horb[5], Paul Coucke[4], Andy Willaert[4], Aaron M. Zorn[3], Kris Vleminckx[1,2,4§]

[1] Department of Biomedical Molecular Biology, Ghent University, Ghent, Belgium

[2] Cancer Research Institute Ghent, Ghent, Belgium

[3] Division of Developmental Biology, Perinatal Institute, and Center for Stem Cell and Organoid Medicine (CuSTOM), Cincinnati Children's Hospital, Cincinnati, USA

[4] Center for Medical Genetics, Department of Biomolecular Medicine, Ghent University, Ghent, Belgium

[5] National Xenopus Resource and Eugene Bell Center for Regenerative Biology and Tissue Engineering, Marine Biological Laboratory, Woods Hole, Massachusetts 02543, USA.

[6] These authors contributed equally to the work: Thomas Naert and Dieter Tulkens

[§] Corresponding author

Kris Vleminckx, Ph.D.
Dept. for Biomedical Molecular Biology
Ghent University
Technologiepark 71
B-9052 Ghent (Zwijnaarde)
Tel +32-9-33-13760, Fax +32-9-221 76 73,
E-mail : kris.vleminckx@irc.UGent.be

Supplementary figure 1

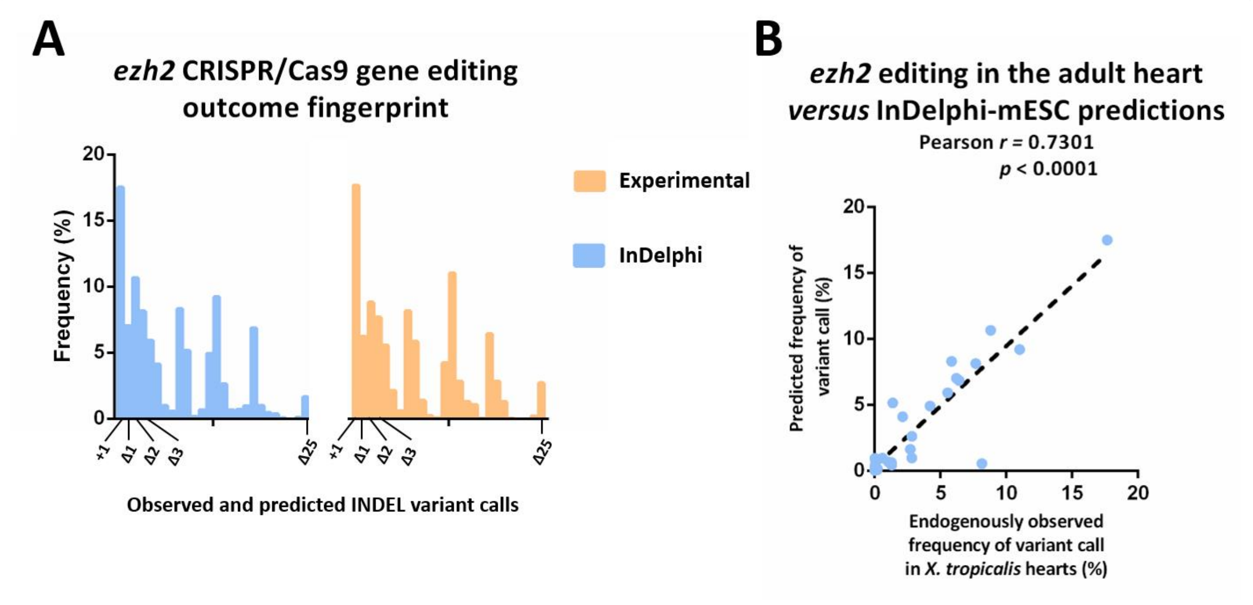

Fig. S1: *ezh2* CRISPR/Cas9 gene editing outcome can be accurately predicted via the online prediction algorithm InDelphi. (A) Column graphs showing overlay of variant calls (%) between *in vivo* observations and *in silico* predictions **(B)** Pearson correlation with significance interval between *in vivo* observations and *in silico* predictions for the ezh2 gRNA.

Supplementary figure 2

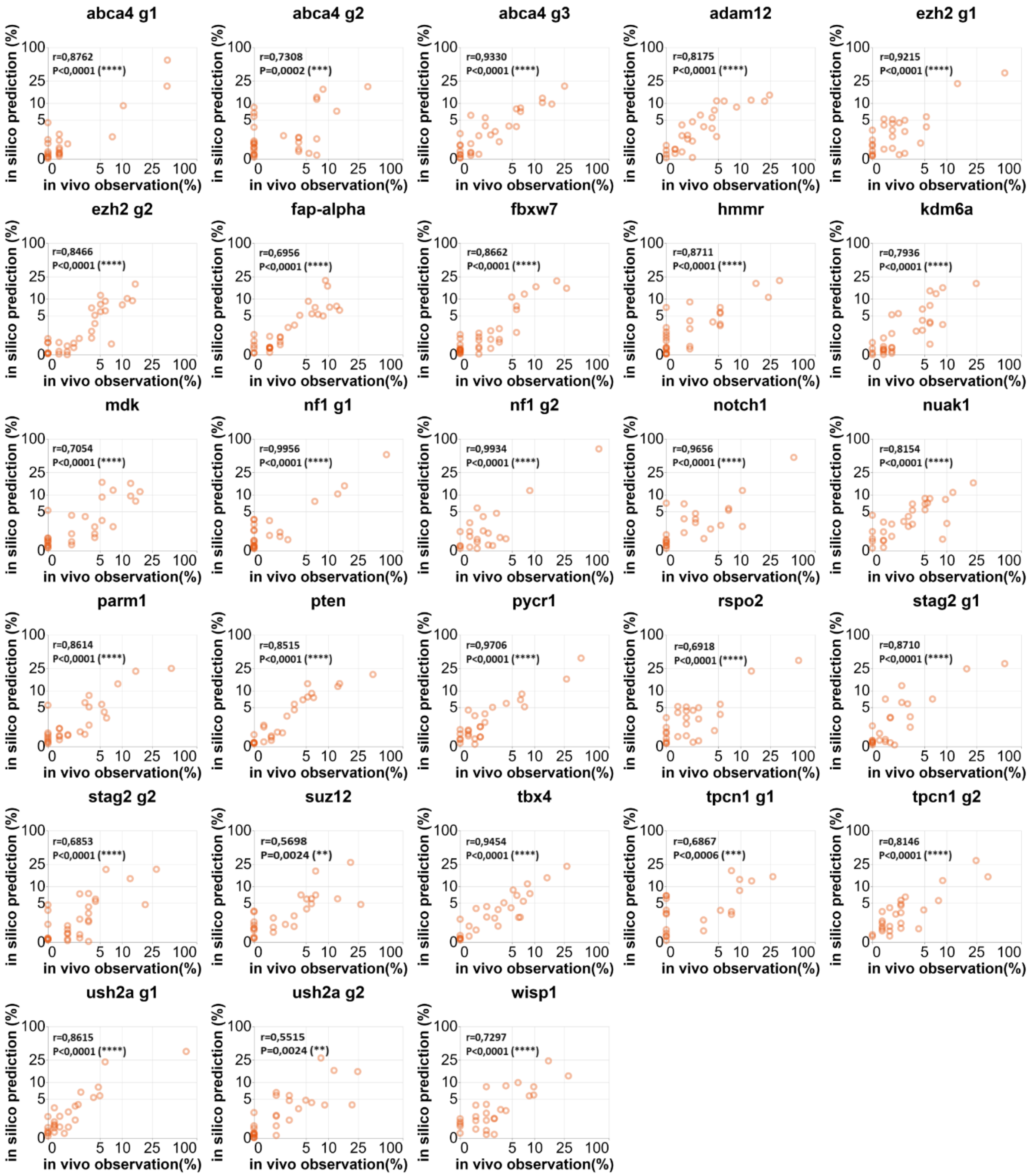

Fig. S2: Pearson correlations between *in vivo* observed (obtained by targeted amplicon sequencing) and respective *in silico* predicted variant frequencies for 28 gRNAs injected in *X. tropicalis* embryos. gRNAs are injected as Cas9/gRNA-ribonucleoprotein complexes at early developmental stages (2 to 8 cell stage). Target regions are PCR amplified and sequenced using MiSeq sequencing (Illumina) and raw data is processed using the BATCH-GE analysis software. *In silico* predictions are generated by the InDelphi software algorithm. Plots show correlations between *in vivo* observed and *in silico* predicted variant frequencies. x_g1, x_g2, x_g3 refers to different guide RNAs against the same gene. (****p < 0.0001; ***p < 0.001; **p < 0.01).

Supplementary figure 3

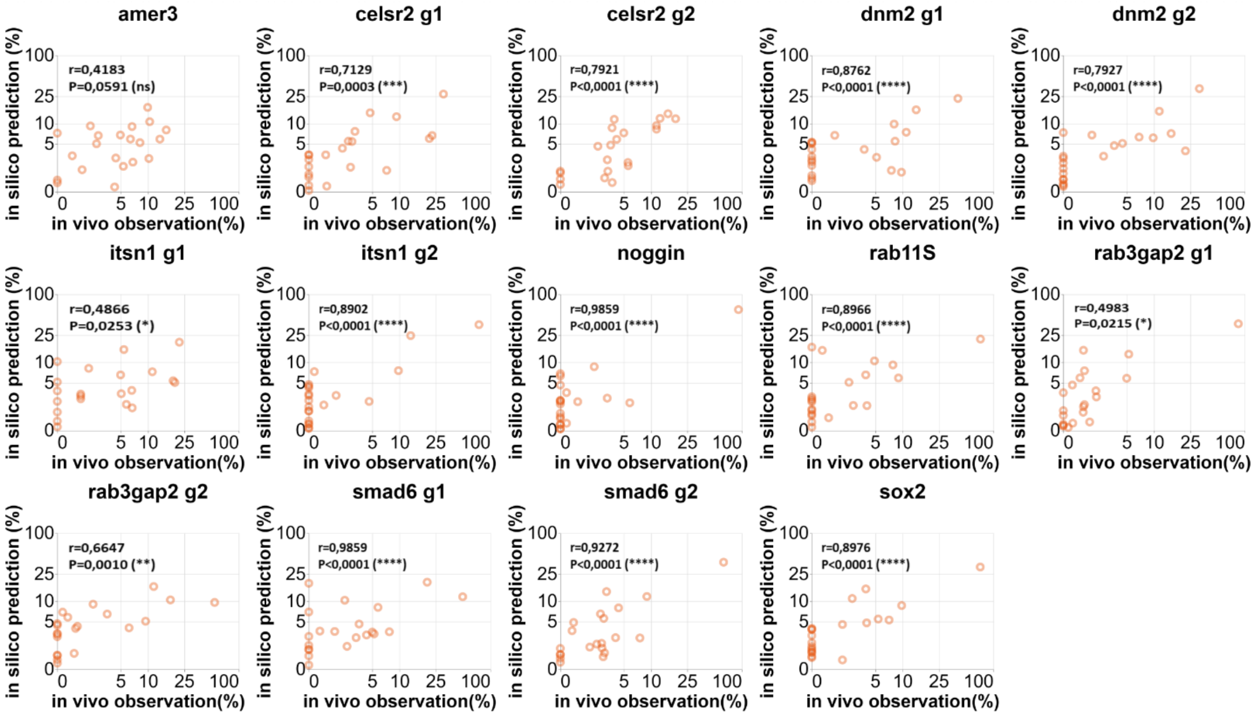

Fig. S3: Pearson correlations between *in vivo* observations (generated by Sanger sequencing and sequence trace deconvolution) and respective *in silico* predictions of 14 gRNAs injected in *X. tropicalis* embryos. gRNAs are injected as Cas9/gRNA-ribonucleoprotein complexes at early developmental stages (1-cell stage). Target regions are PCR amplified and sequenced using Sanger sequencing and deconvoluted using the Inference of CRISPR Edits (ICE) algorithm. *In silico* predictions are generated by the InDelphi software algorithm. Plots show correlations between *in vivo* observed and *in silico* predicted variant frequencies. x_g1, x_g2 refers to different guide RNAs against the same gene. (****p < 0.0001; ***p < 0.001; **p < 0.01; *p < 0.05; ns = not significant).

Supplementary figure 4

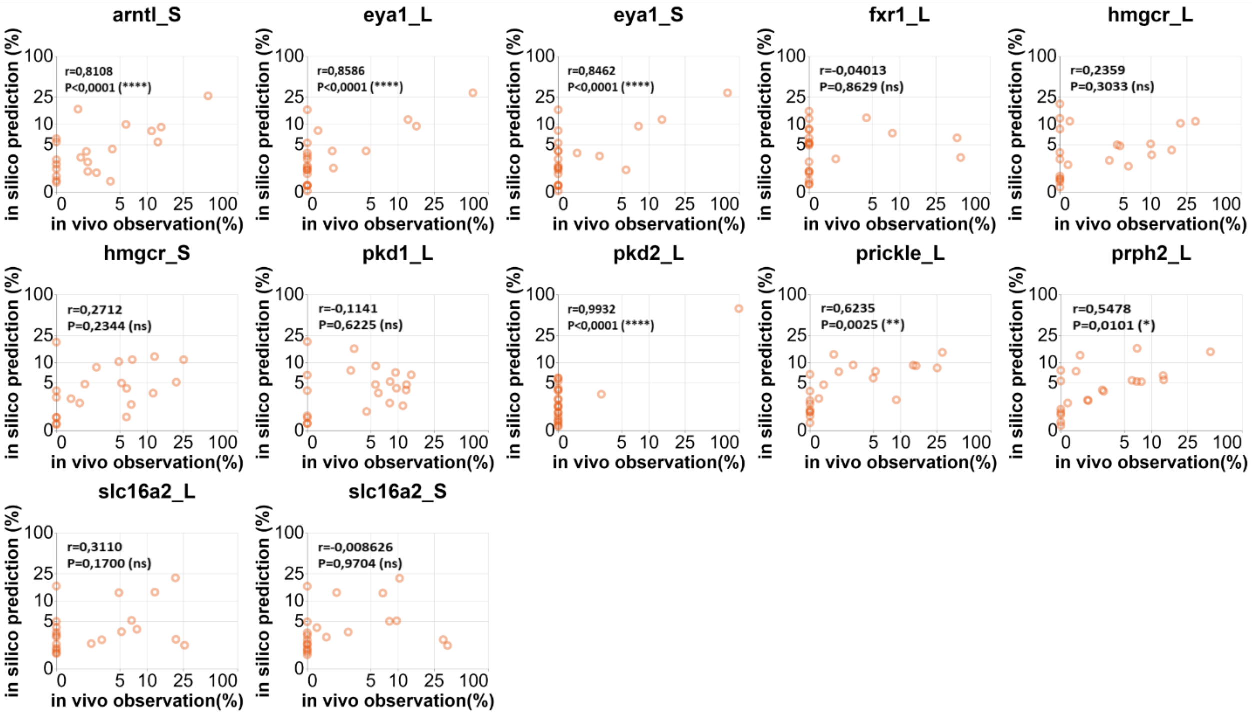

Fig. S4: Pearson correlations between *in vivo* observations (generated by Sanger sequencing and sequence trace deconvolution) and respective *in silico* predictions of 10 gRNAs injected in *X. laevis* embryos. gRNAs are injected as Cas9/gRNA-ribonucleoprotein complexes at early developmental stages (1-cell stage). Target regions are PCR amplified and sequenced using Sanger sequencing and deconvoluted using the Inference of CRISPR Edits (ICE) algorithm. *In silico* predictions are generated by the InDelphi software algorithm. Plots show correlations between *in vivo* observed and *in silico* predicted variant frequencies. Gene name_S and gene name_L refers to the two homeologues of a particular gene present on the small and large chromosome, respectively. (****p < 0.0001; **p < 0.01; *p < 0.05; ns = not significant).

Supplementary figure 5

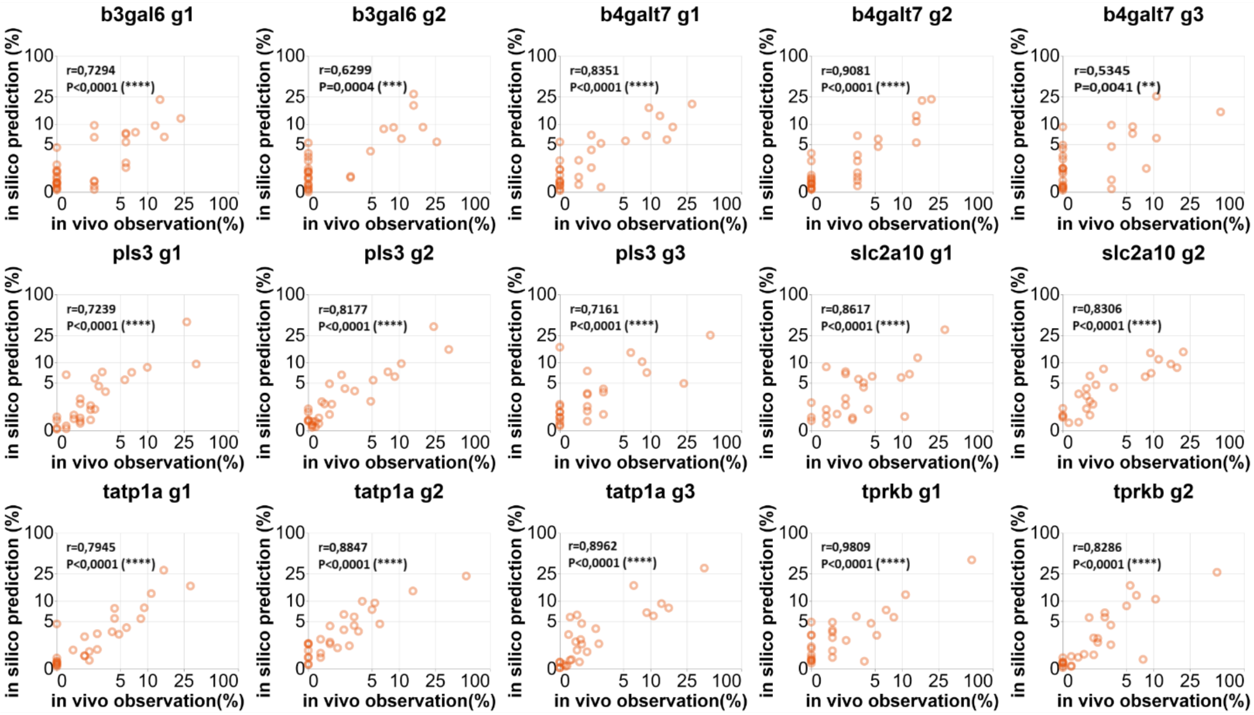

Fig. S5: Pearson correlations between *in vivo* observations (generated by targeted amplicon sequencing) and respective *in silico* predictions of 15 gRNAs injected in zebrafish embryos. gRNAs are injected as Cas9/gRNA-ribonucleoprotein complexes at early developmental stages (1 cell stage). Target regions are PCR amplified and sequenced using MiSeq sequencing (Illumina) and raw data is processed using the BATCH-GE analysis software. *In silico* predictions are generated by the InDelphi software algorithm. Plots show correlations between *in vivo* observed and *in silico* predicted variant frequencies. x_g1, x_g2, x_g3 refers to different guide RNAs against the same gene. (****$p < 0.0001$; ***$p < 0.001$; **$p < 0.01$).

Supplemental figure 6

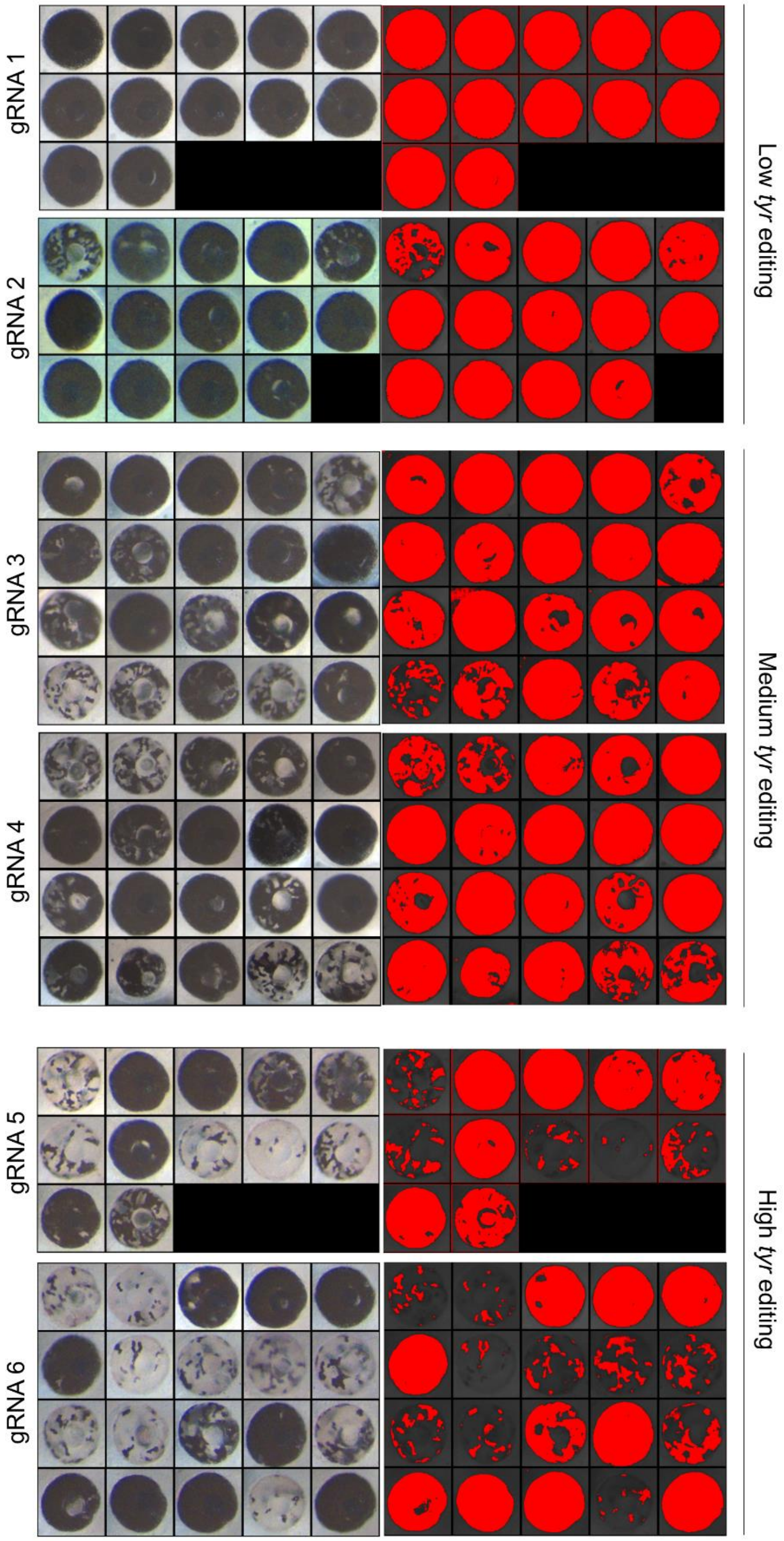

Fig. S6: Pictures from eyes of *tyrosinase* mutant embryos with their associated threshold mask used for quantification.

Supplementary table legends

Supplementary table 1. (Sheet A) *X. tropicalis* guide RNA (n=42) genomic target sites and oligos and genotyping primers for downstream targeted amplicon sequencing. **(Sheet B)** *X. laevis* guide RNA (n=12) genomic target sites, oligos and genotyping primers for downstream Sanger sequencing and sequence trace deconvolution. **(Sheets C)** Zebrafish guide RNA (n=15) genomic target sites and oligos and genotyping primers for downstream targeted amplicon sequencing.

Supplementary table 2. Statistical analyses. In relation to Fig. 2C. Tests of normality (Shapiro-Wilk) show $p>0.05$ for all groups. Significant differences in homogeneity are observed (Levene $p<0.05$). Groups show statistical significant differences (One-way Welsh ANOVA to adjust for unequal variances, $p<0.001$). Games-Howell multiple comparisons are used as post-hoc tests between groups. In relation to Figure 4B. Mann Whitney test reveals statistically significant differences in percentage of repair by MMEJ when respectively comparing highest-in-class gRNAs (n=4,860) to a random selection of gRNAs (n=4,860) ($p<0.001$) and when comparing lowest-in-class gRNAs (n=4,860) to a random selection of gRNAs (n=4,860) ($p<0.001$).

Supplementary table 3. This file contains an overview of the cutting efficiencies of the *tyr* CRISPR gRNAs as determined by PCR amplification and targeted amplicon sequencing.
